# The Evolving Narrative of Black Men’s Brain Health: Implications for Dementia Research and Public Engagement

**DOI:** 10.1093/geroni/igaf122.973

**Published:** 2025-12-31

**Authors:** Darlingtina Esiaka

**Affiliations:** University of Kentucky - College of Medicine, Lexington, Kentucky, United States

## Abstract

This paper considers the concept of “brain health”—the optimal state of brain functioning, encompassing cognitive, sensory, social-emotional, behavioral, and motor aspects -- as it has been deployed as an empowering narrative within the broader discourse of “healthy aging,” emphasizing individual agency and preventative approaches to maintaining cognitive function and reduce dementia risk. Drawing on community-based participatory research and examples from community health initiative, the author considers the tension between this empowering narrative and the stark risk of dementia and precursor conditions within Black communities, focusing on Black men. The author demonstrates the need for narratives about brain health to recognize that Black men’s brain health outcomes are not solely the result of individual choices but are deeply influenced by historical and ongoing inequities. The author explains current efforts to construct a new, strength-based narrative about brain health that can empower and support Black men.

